# Insights into the Effect of Lithium Doping on the Deep Eutectic Solvent Choline Chloride:Urea

**DOI:** 10.3390/ma15217459

**Published:** 2022-10-24

**Authors:** Giselle de Araujo Lima e Souza, Maria Enrica Di Pietro, Franca Castiglione, Valeria Vanoli, Andrea Mele

**Affiliations:** 1 Department of Chemistry, Materials and Chemical Engineering “G. Natta”, Politecnico di Milano, Piazza L. da Vinci 32, 20133 Milan, Italy; 2 CNR-SCITEC Istituto di Scienze e Tecnologie Chimiche, Via A. Corti 12, 20133 Milan, Italy

**Keywords:** deep eutectic electrolytes, nuclear magnetic resonance, lithium salt, correlation time, dynamics, lithium transport

## Abstract

Choline-based deep eutectic solvents (DESs) are potential candidates to replace flammable organic solvent electrolytes in lithium-ion batteries (LIBs). The effect of the addition of a lithium salt on the structure and dynamics of the material needs to be clarified before it enters the battery. Here, the archetypical DES choline chloride:urea at 1:2 mole fraction has been added with lithium chloride at two different concentrations and the effect of the additional cation has been evaluated with respect to the non-doped system via multinuclear NMR techniques. ^1^H and ^7^Li spin-lattice relaxation times and diffusion coefficients have been measured between 298 K and 373 K and revealed a decrease in both rotational and translational mobility of the species after LiCl doping at a given temperature. Temperature dependent ^35^Cl linewidths reflect the viscosity increase upon LiCl addition, yet keep track of the lithium complexation. Quantitative indicators such as correlation times and activation energies give indirect insights into the intermolecular interactions of the mixtures, while lithium single-jump distance and transference number shed light into the lithium transport, being then of help in the design of future DES electrolytes.

## 1. Introduction

The ever-increasing global energy demand is constantly asking for the development of new safe, cheap and environment-friendly energy storage devices. Among the electrochemical energy storage systems, lithium ion batteries (LIBs) represent one of the most successful technologies with a dominant influence on the modern society in a plethora of applications from portable electronic devices, to medical technology, electric and hybrid vehicles, and stationary storage [1,2,3]. However, when subjected to thermal, mechanical and electrical abuses, LIBs can abruptly release chemical energy in the form of fire or explosions, and possible leakage of hazardous chemicals, leading to disastrous accidents involving different devices from cell phones and laptops, to electric vehicles and airplanes [1]. In such uncontrollable thermal runaway processes, liquid electrolytes play a significant role. Currently, state-of-the-art electrolytes in LIBs are composed of a thermally unstable mostly fluorinated lithium salt (usually lithium hexafluorophosphate, LiPF_6_) dissolved in highly flammable organic solvents such as ethylene carbonate, dimethyl carbonate, propylene carbonate and diethylcarbonate [1,2,3]. These electrolytes act as the “fuel” for the battery combustion and explosion in case of a thermal runaway process, and cause in general adverse environmental impacts at the end of the life of the batteries [1,3]. Such concerns over the safety hazard, waste management and environmental impact of LIBs are currently driving the academic and industrial communities to consider alternative non-flammable and environmentally friendly solvents as replacement of the organic electrolytes.

Deep Eutectic Systems (DESs) stand out as a promising option in this respect [4,5]. They inherit the appreciable ionic conductivity and low- or non-flammability of the similar compounds room temperature ionic liquids (ILs) [6], but do not suffer from the same downsides, i.e., high cost, quite complicated synthesis and questioned safety. On the contrary, DESs are endowed with additional advantages such as air and moisture stability, higher solubility for metal salts and wider tunability of functional properties. Besides, DESs are composed of largely available, environmentally friendly and safe raw materials, and they are also easy to prepare with a 100% atom economy and no need for purification steps [4,5]. The cost-efficiency and high sustainability of DESs make them extremely interesting as benign-by-design electrolyte materials in energy storage devices, and promising candidates to replace organic solvents in LIBs.

Although a strict and widely recognized definition does not exist at present, DESs usually consist of a mixture of at least two components, normally solid at room temperature, which self-associate when mixed at a given mole ratio, with formation of a “deep” eutectic mixture. The establishment of intermolecular interactions, mainly–but not only–hydrogen bonds (H-bonds), results in a marked depression of the melting point around the eutectic point with respect to both the pure constituents and the one predicted by assuming a thermodynamic ideal behavior of the solid-liquid equilibrium [4,7].

Among the different families of DESs (Table S1) [8,9], type IV DESs (mixtures of a metal salt and a molecular hydrogen bond donor, HBD) dominate the electrochemical field, while only a few applications of type III DESs (composed of an organic salt and a molecular HBD) are reported [5,10,11]. The claimed advantage of the former (and vice versa drawback of the latter) would be the presence of Li^+^ as single cation species in the mixture, thus enabling the achievement of higher transference numbers [11,12,13]. It should be noted, however, that preparing those type IV DESs requires large amounts of expensive lithium salt, rising the production costs when compared to dissolving controlled amounts of the same salt in low-cost type III DESs, hence jeopardizing one of the merits of DESs. In a previous work [14], we studied a prototypical system composed of choline chloride:urea at 1:2 mole fraction (ChCl:U x_ChCl_ = 0.33) with LiCl dissolved at a concentration of 0.4 mol/kg. A combination of multinuclear NMR measurements and molecular dynamics simulations pointed towards a strong coordination of Li^+^ cations by chloride anions and a marked involvement of U in the H-bond network. These preliminary results suggest a systematic evaluation of the effect of lithium doping in the underexploited type III DESs electrolyte solutions. Actually, the addition of a small positively charged ion, Li^+^, is known to affect both the macroscopic (e.g., viscosity [15]) and the microscopic (e.g., molecular diffusion [16]) properties of ChCl. Since the peculiar macro- and microscopic behavior of DESs stems from a tangled–and often not trivially predictable–balance of intermolecular forces, this calls for a profound understanding, at molecular /atomic level, of the effect of the addition of lithium ions in such network, for the design, optimization, and development of deep eutectic electrolytes (DEEs).

In the present work, we contribute to the experimental design mentioned above by increasing lithium contents (0.8 and 1.0 mol/kg) and comparing the Li-doped systems with the pristine Li-free ChCl:U. This allows to gather more insights into the effect of Li-doping and Li-concentration on the dynamic behavior of the different species in the mixtures. ^35^Cl NMR spectra were collected over a 75 K temperature interval in 5 K temperature steps, with the linewidths reflecting both the viscosity increase after LiCl addition and the underlying Li-Cl complexation. ^1^H and ^7^Li spin-lattice relaxation and diffusion measurements were performed as a function of temperature, and interpreted in terms of correlation times, rotational and translational activation energies, transference number and lithium single-jump distance.

## 2. Materials and Methods

All materials, choline chloride (ChCl, ≥ 98%), urea (U, ≥ 98%) and lithium chloride (LiCl, ≥99%) were purchased from Sigma Aldrich and vacuum dried prior to use. The DES ChCl:U at 1:2 molar ratio (x_ChCl_ = 0.33) was prepared by mixing the two components, ChCl and U, at the proper mole fractions and heating at 80 °C under constant stirring until the formation of a homogeneous liquid. Next to the pure DES (LiCl 0 wt%), two lithium-doped samples were prepared by adding LiCl to the mixture under stirring at two different concentrations (ca. 3 wt% and 4 wt%). The water content of the samples was below 2 wt% as measured by Karl-Fischer titration. A summary of the samples is given in Table 1, while the structure of the components is shown in Figure S1.

The samples were transferred to 5 mm NMR tubes, equipped with a capillary containing deuterated dimethylsulfoxide (DMSO-d_6_). NMR measurements were performed without sample spinning with a Bruker NEO 500 console (11.74 T) equipped with a direct observe BBFO (broadband including fluorine) iProbe and a variable-temperature unit (^1^H, ^7^Li and ^35^Cl resonance frequency of 500.13, 194.37 and 49.00 MHz, respectively). The instrument was carefully tuned, shimmed, and the 90° pulses calibrated.

1D ^1^H, ^7^Li and ^35^Cl, as well as ^1^H and ^7^Li T_1_ relaxation and diffusion experiments were performed over a temperature range of 298 K to 373 K, in 5 K increments, with a minimum of 15 min allowed for thermal equilibration. 1D ^1^H and ^7^Li spectra were recorded with 1 scan using 16384 points, over a spectral width of 9 and 50 ppm, respectively. ^35^Cl spectra were collected with standard 1D acquisition sequences, using the ARING pulse train to reduce acoustic ringing artefacts in ^35^Cl measurements. Automated peak-picking and linewidth routines were used for estimations of ^35^Cl full width at half maximum (FWHM).

^1^H and ^7^Li T_1_ relaxation measurements were carried out with the inversion recovery (IR) pulse sequence. Spectra were recorded using relaxation delays at least five times the longest T_1_, four dummy scans prior to acquisition, and data matrices of 16,384 (t_2_) × 16 (t_1_), with 2 transients per increment, over a spectral width of 9 ppm for ^1^H and 50 ppm for ^7^Li. The spin−lattice relaxation rates were measured for delay times ranging from 0.05–5 s to 0.05–10 s, according to the temperature. The baselines of all arrayed T_1_ spectra were corrected prior to processing the data using an exponential filter in F_2_ dimension (with LB equal to 0.3 Hz). Relaxation times were computed using integrals from experimental raw data by means of the Bruker T_1_/T_2_ relaxation module using the manual integration option and applying the standard one-component fitting function. Fits to extract the correlation times and rotational activation energies from the relaxation rates were performed with OriginPro 2018 using a user-defined function with the Levenberg–Marquardt algorithm.

^1^H and ^7^Li self-diffusion coefficients were measured by pulsed field gradient (PFG)-NMR experiments by applying sine-shaped pulsed magnetic field gradients along the z-direction up to a maximum strength of G = 48.15 G cm^−1^. The diffusion experiments were performed using the bipolar pulse longitudinal eddy current delay (BPP-LED) pulse sequence. All experiments were carried out using 16384 points in the F_2_ dimension, over a spectral width of 9 ppm for ^1^H and 50 ppm for ^7^Li, with a total of eight transients per increment. The relaxation delay was set to at least five times T_1_, and four dummy scans were programmed prior to acquisition. The pulse gradients were incremented from 2 to 95% of the maximum gradient strength in a linear ramp with 32 steps. For each DOSY experiment, the duration of the magnetic field pulse gradients (δ) and the diffusion times (Δ) were optimized to obtain, where possible, 95% signal attenuation for the slowest diffusion species in the last step experiment. For ^1^H experiments, δ values were in the 2.6–6.0 ms range, while Δ values were 0.3–0.8 s long. For ^7^Li experiments, δ and Δ values were equal to 6.0 ms and 0.8 s, respectively. Due to slow motion, not enough signal attenuation was achieved at the lowest temperatures, then diffusion data for lithium were collected in the reduced temperature range 318–373 K. The baselines of all arrayed spectra were corrected prior to processing the data. Data were processed using an exponential filter in F_2_ dimension (LB = 0.3 Hz), and integrals were used in calculating relaxation times. The Stejskal-Tanner equation [17] was used to extract the self-diffusion coefficients for each peak via the Bruker T_1_/T_2_ module of TopSpin. Linear fits of the Arrhenius plots were performed with OriginPro 2018.

## 3. Results and Discussion

At the light of the results obtained on archetypal lithium-doped ChCl:U [14], here we propose a more systematic investigation on two samples containing a higher amount of metal salt (0.8 and 1.0 mol/kg), and compare the results with the pure DES. This allows to gather novel insights into the effect of Li-doping and Li-concentration on the dynamic behavior of the different species in the mixtures. Note that ChCl and most type III DESs are highly hygroscopic, hence preventing the preparation of a water-free system when working under ambient conditions. We found previously that a small amount of water (3.9 wt%) substantially does not alter the intermolecular structure of the ChCl DES electrolyte, but has a remarkable effect on dynamics [14]. This is in line with the by now established concept that a controlled amount of water does not seriously break up the intermolecular network of neat DESs [18], but has a non-negligible effect in reducing viscosity. Due to preliminary vacuum drying, a residual amount of water (2 wt%) must be considered here for the three samples, which is close to a realistic scenario for industrial processes carried out at ambient air conditions.

Although, when designing a DEE, the obvious interest is focused on lithium, the study of its local environment and mobility cannot leave aside all other components of the mixtures. The NMR approach applied in the present work allows from the one hand to probe individually the rotational and translational mobility of the different species and on the other to gain an overall picture of the intermolecular network within the mixtures. In this way, we gain a deeper understanding of how the strength of the intermolecular interactions in the DEE change at increasing lithium concentration and in comparison to the Li-free system. We discuss in the following the results obtained from chemical shift/linewidth analysis, relaxation and diffusion measurements on the different species in the mixtures, with a special focus on quantitative descriptors of lithium transport.

### 3.1. 1D ^1^H, ^7^Li and ^35^Cl NMR: Temperature-Dependent Chemical Shift and Linewidth

**Figure 1:**
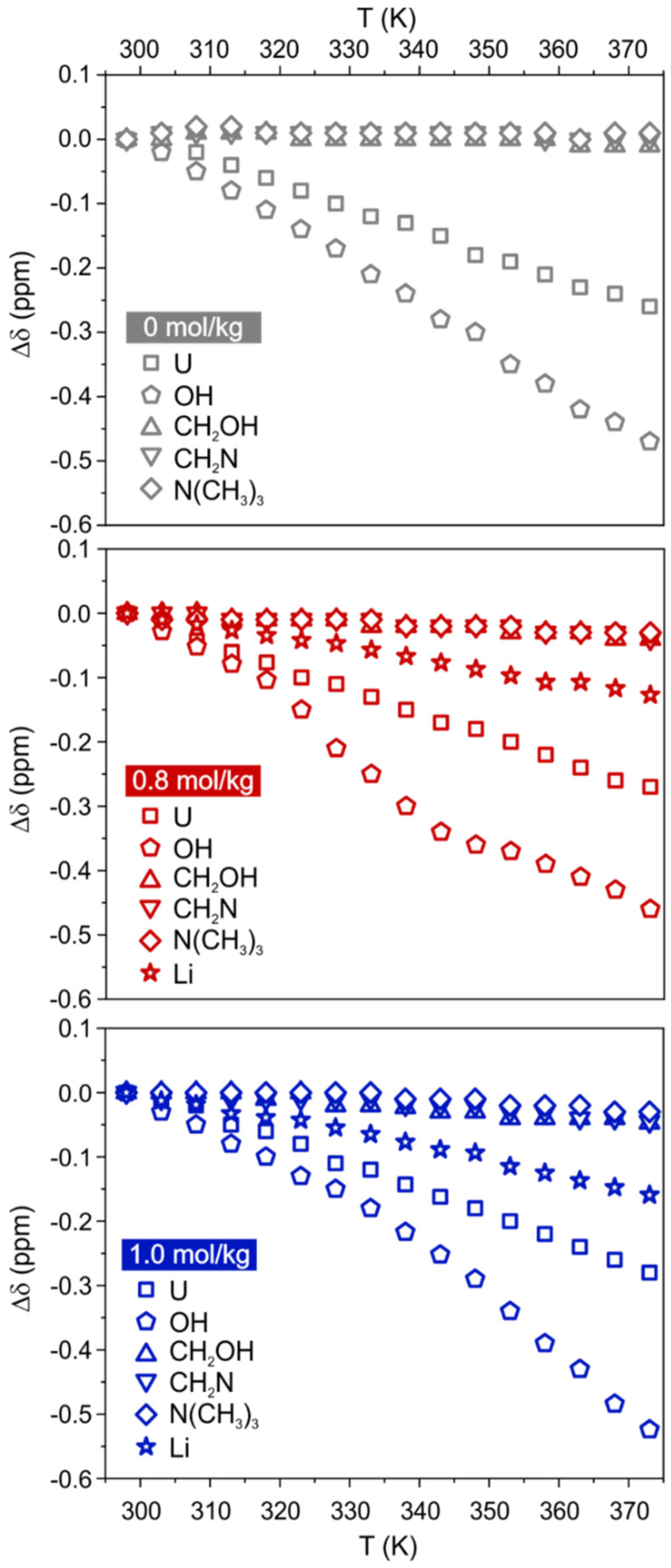
^1^H and ^7^Li chemical shift variations of ChCl:U-LiCl(0) (top, grey), ChCl:U-LiCl(0.8) (middle, red), and ChCl:U-LiCl(1.0) (bottom, blue), as a function of temperature. Maximum errors are estimated to be 2% for ^1^H and ^7^Li, and 4% for OH protons.

The analysis of chemical shift variations gives preliminary hints into the strength of the intermolecular interactions experienced by the different species (Figure 1). As expected, an upfield shift is observed for all sites, indicating a weakening of the interactions due to the increase in temperature. As a result of their major involvement in the H-bond network, the peaks of U and OH are the most sensitive to the temperature change. Choline signals are barely affected, while Li shows a moderate upfield shift. Noteworthy is that the addition of Li salt does not have a drastic impact on the temperature-dependent ^1^H chemical shifts (for instance, $\left|\delta \left(373\;K\right)-\delta \left(298\;K\right)\right|=\left|\Delta \delta^{max}\right|$ is in the range 0.3–0.5 ppm for U and OH, respectively, in all samples). The presence of LiCl is instead affecting the linewidth of ^35^Cl signal, causing a significant increase of the full width at half maximum (FWHM) with respect to the neat DES (Figure S2), which is an expected consequence of the viscosity increase. As expected, the increase in temperature causes a marked drop in linewidth of the ^35^Cl signal, likely due to the decrease in viscosity. Perusal of Figure S2b also highlights a change in slope of the curve of ^35^Cl NMR linewidth vs T at about 335 K for the two Li containing samples, while no evident change is observed in the curve related to the DES without LiCl. Thus, the profile of the temperature-dependent FWHMs in the presence of LiCl shows a smaller gradient in the first T region (up to roughly 335 K) with respect to the non-doped system. Given the ^35^Cl linewidth is indicative of the Cl^-^ local environment [19], this would qualitatively indicate that, in the presence of Li^+^, the chloride local environment is less sensitive to the temperature increase–reasonably due to Li complexation. Data for ChCl:U-LiCl(0.8) and ChCl:U-LiCl(1.0) mostly overlap, thus suggesting that both the viscosity and the symmetry of the chloride environment are comparable in the two lithium-doped systems.

### 3.2. ^1^H and ^7^Li PFG-NMR: Self-Diffusion Coefficients, Translational Activation Energy and Transference Number

PFG-NMR was applied to collect the self-diffusion coefficients of the different species in the mixtures over a 75 K temperature interval, using 5 K steps.

**Figure 2:**
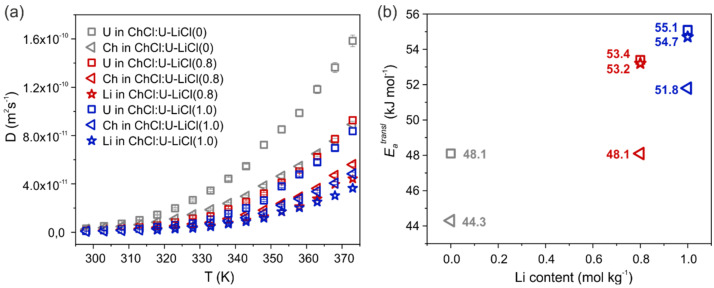
(**a**) Diffusion coefficients obtained for Ch (triangle), U (square) and Li (star) in the samples ChCl:U-LiCl(0) (grey), ChCl:U-LiCl(0.8) (red), and ChCl:U-LiCl(1.0) (blue), as a function of temperature. The self-diffusion of CH_3_ is used here as representative for Ch, all cholinium protons exhibiting the same diffusivity. Maximum errors are estimated to be 3% for ^1^H and 5% for ^7^Li. (**b**) Apparent translational activation energy $E_{a}^{transl}$ obtained from the Arrhenius plots of diffusion data of for Ch (triangle), U (square) and Li (star), as a function of the LiCl content (0 mol/kg = grey, 0.8 mol/kg = red, and 1.0 mol/kg = blue).

Table S2 and Figure 2a show the self-diffusion coefficients measured for the different species in the three samples by PFG-NMR. Diffusivity follows the order U > Ch > Li. Lithium is hence the slowest species in the mixtures, due to the strong interactions involving this cation. Indeed, Li^+^ interacts strongly with chloride anions, as already observed for the parent system with LiCl 0.4 mol/kg [14] and is widely known for similar systems like ionic liquid mixtures [20,21]. An additional contribution to the slow translational dynamics of Li^+^ stems from its interactions with urea in the second coordination shell [14]. The addition of LiCl 0.8 mol/kg roughly halves the diffusion of both U and Ch, which is further slowed down when more LiCl is added. This is intuitively related to the increased viscosity of the system once the metal salt is added.

The apparent activation energies for the translational motion, $E_{a}^{transl}$, were obtained fitting the diffusion data to an Arrhenius expression and are reported in Figure 2b and Table S3. In the two Li-doped samples, $E_{a}^{transl}$ is in the order: Li ≈ U > Ch. The gap in activation energy between Li and Ch seems to be inversely proportional to the LiCl content (2.9 kJ/mol < 5.1 kJ/mol < ca. 7 kJ/mol for concentrations of 1.0 mol/kg, 0.8 mol/kg and 0.4 mol/kg, respectively) [14]. In the previous work [14], we highlighted a peculiar behavior of U, which is showing a diffusion behavior–and consequently a $E_{a}^{transl}$–strongly dependent on its average H-bond interactions in the system, and then on the temperature. In particular, the activation energy of U was found to be well correlated with Li–Cl complexes in the low T regime, and with Ch in the high T regime. Here we observe that the non-Arrhenius behavior of urea diffusivity is even more marked in the pure DES (R^2^ = 0.990), but, on the other hand, it is less relevant in the Li-doped systems (R^2^ = 0.996 and 0.998 for LiCl 0.8 mol/kg and 1.0 mol/kg, respectively). Increased amount of LiCl would reduce the temperature-dependence of U activation energy, making it closer to that of lithium itself in all the investigated temperature range (53.4 vs. 53.2 kJ/mol in ChCl:U-LiCl(0.8) and 55.11 vs. 54.7 kJ/mol in ChCl:U-LiCl(1.0)). Bearing in mind that Li interacts with urea in the second coordination shell [14], this would indicate a strengthening of such interactions with increased Li content.

**Figure 3:**
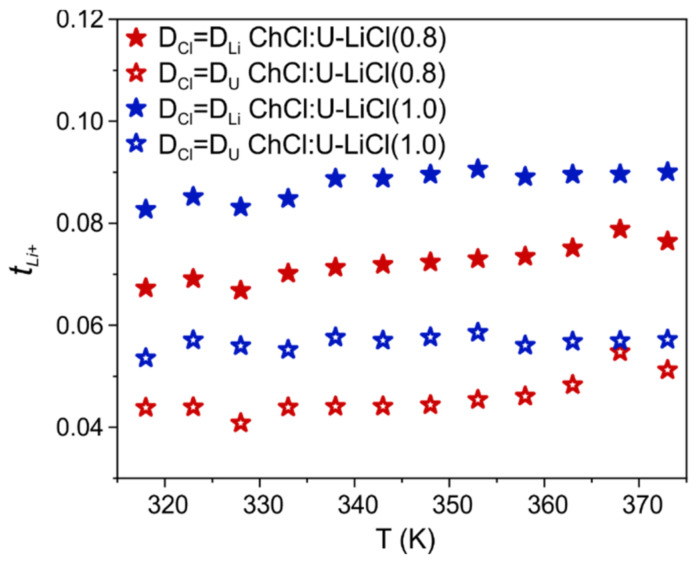
Apparent transference number for lithium ions in the two Li-doped systems (0.8 mol/kg = red, and 1.0 mol/kg = blue), assuming the self-diffusion coefficient of chloride equal to lithium (filled symbols) or urea (empty symbols), as a function of temperature.

A convenient way to quantify the relative mobility of Li ions with respect to other species in the mixture is via the transference number, which is the fractional contribution of an ion species to the overall conductivity [22]. Indeed, all ions in the system contribute to the total current, but Li ion transport is crucial for the charge/discharge process of LIBs [23]. Under the assumption that all ionic species are free, i.e., ion pairs and/or ion clustering are neglected, an apparent transference number for Li can be calculated from NMR diffusion experiments as given by $t_{Li^{+}}=n_{Li^{+}}D_{Li^{+}}/\sum_{i}n_{i}D_{i}$, with $n_{i}$ the number of charge carriers [23]. Given the diffusion coefficient of the chloride anion is not directly accessible by PFG-NMR, a Li ion transference number cannot be calculated. However, previous MD simulations in ChCl:U systems found that chloride diffusivity is intermediate between Ch and U [24], with values closer to Ch in the presence of the additional Li cation [14]. Hence, we can define two boundary conditions for $t_{Li^{+}}$ by considering $D_{Cl}$ equal to the self-diffusion measured for the fastest (U) or the slowest (Li) species (Figure 3), respectively. This gives us the opportunity to estimate the upper and lower transference number of Li ion in this new DES-based electrolyte and compare with other DESs and DES analogs. As observed in Figure 3, estimated $t_{Li^{+}}$ ranges for the two samples from 0.04 to 0.09, with a negligible change when increasing the LiCl content. These values are much smaller than those found for type IV DESs composed of lithium bis(trifluoromethanesulfonyl)imide (LiTFSI) and urea or a sulfonamide as HBD ($t_{Li^{+}}$ between 0.4 and 0.7 in the temperature range 298–353 K) [13,25]. $t_{Li^{+}}$ values between 0.44 and 0.61 at 30 °C were also reported by Watanabe and coworkers in solvated IL systems consisting of equimolar mixtures of a lithium salt and a glyme [12,26]. However, values found here for ChCl:U-LiCl are in line or even higher than transference numbers found for traditional mixtures of ILs and lithium salts (i.e., $t_{Li^{+}}$ in the range 0.02–0.15 are common values for alkyl-imidazolium and alkyl-pyrrolidinium ILs doped with lithium salts [27,28,29]). Therefore, even bearing in mind that apparent transference numbers are only an approximate concept that does not take into account correlated motion of ions, the values found here for the prototypical ChCl:U-LiCl system are probably too low for battery applications at present. The high tunability of DES offers, however, large room for improvement and justify–or even push–further investigations on developing other DEEs.

### 3.3. ^1^H and ^7^Li Relaxation NMR: Correlation Times, Rotational Activation Energy and Lithium Single-Jump Distance

**Figure 4:**
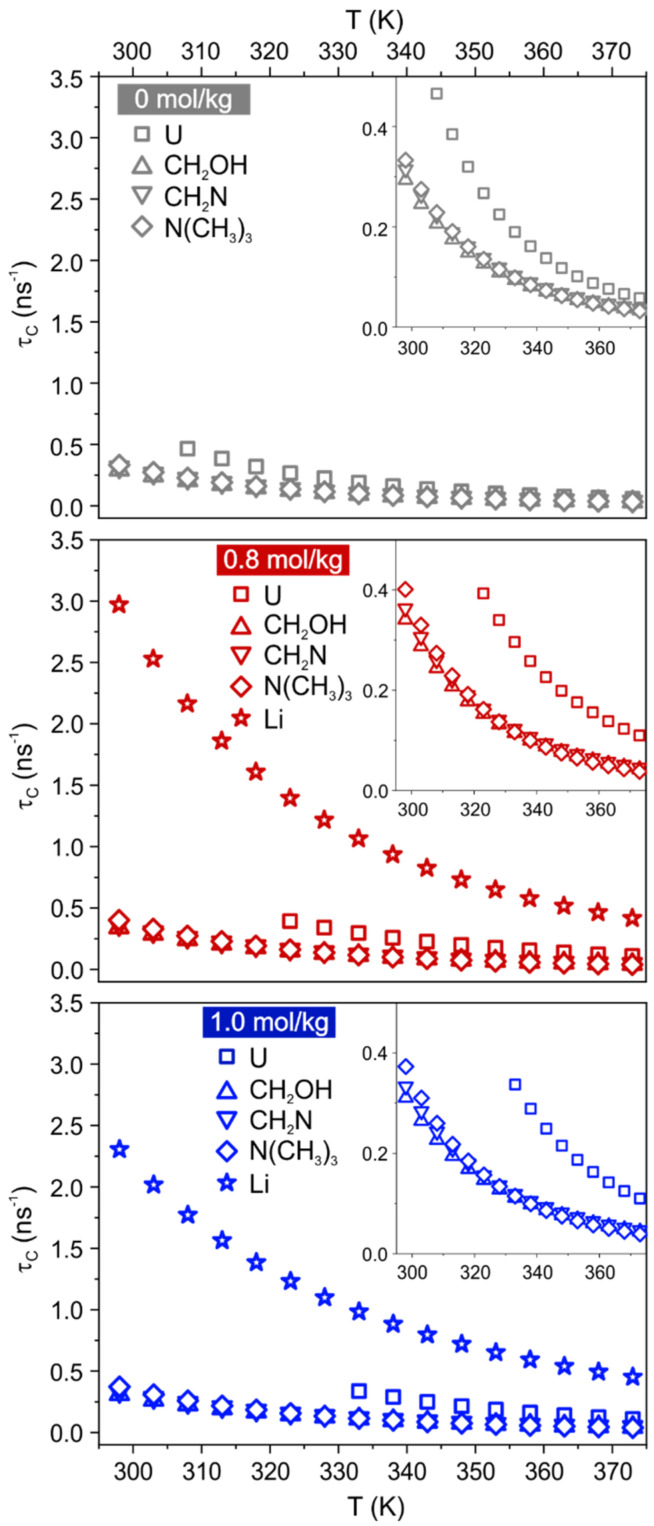
^1^H and ^7^Li correlation time $\tau_{C}$ calculated as a function of temperature in the samples ChCl:U-LiCl(0) (top, grey), ChCl:U-LiCl(0.8) (middle, red), and ChCl:U-LiCl(1.0) (bottom, blue).

^1^H and ^7^Li T_1_ relaxation times were measured between 298 K to 373 K (Figure S3). Curves of Ch protons pass through a minimum at lower temperature than U protons, while Li curves approach a minimum without reaching it. This is a first indication that Ch protons experience faster local dynamics compared to U and Li. To quantify the rotational motion at each site/species, the Bloembergen, Purcell, and Pound (BPP) model [14,21,30,31,32,33,34,35] was applied to the spin-lattice temperature-dependent relaxation data. The obtained correlation time $\tau_{C}$ and apparent activation energy for the rotational motion $E_{a}^{rot}$ are reported in Figure 4 and Tables S4–S7. As already observed [14], the T_1_ curve of U is not reproduced by the BPP model at low temperature, and the divergence gets more pronounced as the LiCl content increases. A good fit of T_1_ data for urea could be obtained in the interval 308–373 K, 323–373 K and 333–373 K for samples ChCl:U-LiCl(0), ChCl:U-LiCl(0.8) and ChCl:U-LiCl(1.0), respectively. Note, however, that no significant differences are observed in the output parameters when fitting data for ChCl:U-LiCl(0) and ChCl:U-LiCl(0.8) in the reduced interval used for ChCl:U-LiCl(1.0) (Table S4).

Applying the BPP equation for the dominant ^1^H-^1^H dipolar relaxation mechanism, the correlation time of the dipolar interaction $\tau_{C}\left(H\right)$ can be obtained [21,31,32,36]. $\tau_{C}\left(H\right)$ can be thought of as the time for a molecular rotation by roughly 1 radian about any axis [37]. Note that for small rigid molecules, it is representative of the whole molecular reorientational time constant [38], whereas for flexible molecules it contains also contributions from internal motions of each given segment [31,32,39]. In both Li-free and Li-doped systems, Ch protons have shorter $\tau_{C}\left(H\right)$–hence faster rotational motion–than U protons, in agreement with previous observations [14,31] and with the considerations drawn before. The addition of Li salt 0.8 mol/kg causes an increase in all $\tau_{C}\left(H\right)$ compared to the neat DES (see also Figure S4 in the ESI). For instance at 333 K, $\tau_{C}\left(H\right)$ values of Ch aliphatic protons slightly increase from 95–100 ps in ChCl:U-LiCl(0) to 115–125 ps in ChCl:U-LiCl(0.8), and $\tau_{C}\left(H\right)$ values of U protons from 0.19 ns in ChCl:U-LiCl(0) to 0.30 ns in ChCl:U-LiCl(0.8). This indicates an overall slowdown of the rotational motion of all sites, which is compatible with a macroscopic increase of viscosity of the system. A further addition of LiCl does not have a marked effect, but it seems to affect differently urea and cholinium: while $\tau_{C}\left(H\right)$ values of U protons slightly increase in ChCl:U-LiCl(1.0) (0.34 ns at 333 K), $\tau_{C}\left(H\right)$ values of Ch protons slightly decrease (112–120 ps at 333 K). Even if interpreted with care, this different behavior would support again a scenario where interactions with U are strengthened on going from 0.8 mol/kg to 1.0 mol/kg of LiCl, while Ch protons are not strongly involved in the network.

**Figure 5:**
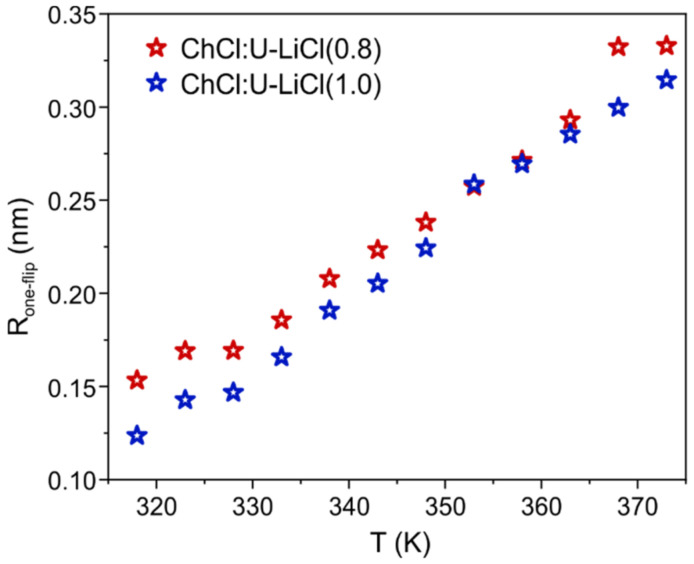
Average single-jump distance for a lithium ion in the two Li-doped systems (0.8 mol/kg = red, and 1.0 mol/kg = blue), as a function of temperature.

To give a physical interpretation to $\tau_{C}\left(Li\right),$ it has to be considered that the dominant relaxation mechanism is represented here by the quadrupolar interaction. Such interaction arises from the coupling of the electric quadrupole moment –a property of the nucleus– with an electric field gradient (EFG), which is intrinsic to the sample [40]. As $\tau_{C}\left(Li\right)$ reflects the fluctuations of the EFG, and given that the EFG is generated by the surroundings, a reasonable interpretation of $\tau_{C}\left(Li\right)$ is less intuitive than $\tau_{C}\left(H\right)$ and has to take into account both the local environment of the ion and the deformation of the electronic cloud of the ion itself induced by the surroundings [40]. A simplified model assumes that the first solvation shell plays the dominant role in the fluctuations of the EFG at the nucleus site and, in the specific case of the Li, oscillations of the EFG correspond to the moving of this ion in its solvation cage [41]. In this description, ^7^Li relaxation would occur by the quadrupolar mechanism for translational motion rather than rotational motion of lithium atom [34,35], and $\tau_{C}\left(Li\right)$ would represent a single lithium jump from one position to another. This allows one to calculate an average distance for a Li one-jump as $R_{one-flip}=\sqrt{6D\tau_{C}\left(Li\right)}$ [34,35]. The estimated average jump distances for the two Li-doped systems investigated here are displayed in Figure 5. $R_{one-flip}$ values range between 0.10 and 0.35 nm in the temperature window 318–373 K, hence slightly smaller than what previously calculated for the more hydrated parent system comparing the same temperature range [14]. However, the overall similarity of the trends would suggest an analogous Li transport within the mixture, i.e., lithium ions migrating via jumps within the solvation sphere.

## 4. Conclusions

Deep eutectic electrolytes show high attractiveness and competitiveness with respect to organic electrolytes and ILs and hold great promise for LIB applications. However, compared to the former cases, which are substantially mixtures of a solvent and a lithium salt, a rationalization of Li^+^ solvation and transport is even more difficult to achieve in the case of DES-based Li electrolytes, which are characterized by a higher system complexity due to both the higher number of actors and the variety of intermolecular interactions among them. Urea’s dynamic behavior emerged as paradigmatic: diffusion data showed that the participation of U to the network of interactions is not only temperature-dependent but also LiCl concentration-dependent. This adds fundamental knowledge to the type of intermolecular interactions taking place in the solvation shells and opens the possibility of modulating the transport properties of the system. In general, the prototypical systems ChCl:U-LiCl studied here rise interest not only for possible applications, but also as probes for exploring the effect of Li-doping on the structure and dynamics of the different species in the mixtures. Multinuclear NMR allows to monitor–at atomic level–the dynamics of the single components. Additionally, the different dominant mechanisms of nuclear relaxation (dipolar and quadrupolar) previously discussed allow to draw a picture of the rotational and translational dynamics of the single components. In the case of ^7^Li relaxation, the dominant “jump” mechanism of diffusion outlined here provides fundamental information for DEE design. All considered, the picture emerging from this study shows high tunability of DEEs and exceptional dissolution abilities, which are the elements to overcome the low mobility of the Li^+^ cation found, a potential showstopper for future applications.

## Figures and Tables

**Table 1:** Samples used in this work.

Sample	LiCl Content (mol/kg)	LiCl Content (wt%)
ChCl:U-LiCl(0)	0	0
ChCl:U-LiCl(0.8)	0.8	3.2
ChCl:U-LiCl(1.0)	1.0	3.9

## Supplementary Materials

The following supporting information can be downloaded at: https://www.mdpi.com/article/10.3390/ma15217459/s1, Figure S1: Chemical structures; Figure S2: ^35^Cl NMR full width at half maximum; Figure S3: ^1^H and ^7^Li T_1_ relaxation times; Figure S4: ^1^H correlation time. Table S1: The main types of DESs; Table S2: Temperature-dependent ^1^H and ^7^Li self-diffusion coefficients; Table S3: Parameters of the linear fit of the Arrhenius plot of the diffusion data; Table S4: Best-fit parameters of the BPP model; Tables S5–S7: ^1^H and ^7^Li rotational correlation times. Reference [8,9] are cited in Supplementary Materials.
