# Supplementary material for: Insights into the Effect of Lithium Doping on the Deep Eutectic Solvent Choline Chloride:Urea
Source: Materials (Basel). 2022 Oct 24;15(21):7459. doi: 10.3390/ma15217459 (PMC9656420; doi:10.3390/ma15217459)
Supplement: Supplementary file 1 [file materials-15-07459-s001.zip › materials-1947615-supplementary.pdf]

materials

Electronic Supporting Information for:

Insights into the effect of lithium doping on the deep eutectic solvent choline chloride: urea

Giselle de Araujo Lima e Souza [1], Maria Enrica Di Pietro [1,*], Franca Castiglione [1], Valeria Vanoli [1], Andrea Mele [1,2,*]

[1] Department of Chemistry, Materials and Chemical Engineering "G. Natta", Politecnico di Milano, Piazza L. da Vinci 32, 20133 Milano, Italy
[2] CNR-SCITEC Istituto di Scienze e Tecnologie Chimiche, Via A. Corti 12, 20133 Milano, Italy
* Correspondence: mariaenrica.dipietro@polimi.it (M.E.D.P.); andrea.mele@polimi.it (A.M.)

Table S1. The main types of DESs [1,2].

Type	General composition	Example
I	Metal salt + organic salt	$ZnCl_2$ + ChCl
II	Metal salt hydrate + organic salt	$CoCl_2 \cdot 6\ H_2O$ + ChCl
III	Organic salt + Hydrogen Bond Donor	ChCl + U
IV	Metal salt (hydrate) + Hydrogen Bond Donor	$ZnCl_2$ + U
V	Molecular non-ionic Hydrogen Bond Donor and Acceptor	thymol + menthol

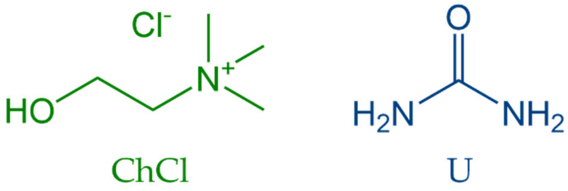

Figure S1. Chemical structure of choline chloride (ChCl) and urea (U).

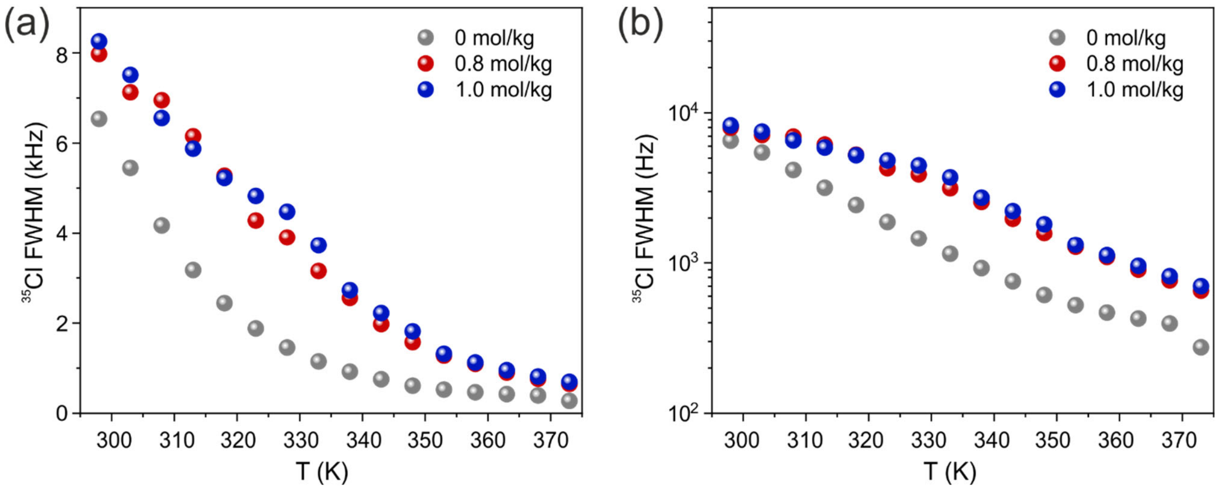

Figure S2. ^{35}Cl NMR (a) linear and (b) log-scaled full width at half maximum (FWHM) of ChCl:U-LiCl(0) (grey), ChCl:U-LiCl(0.8) (red), and ChCl:U-LiCl(1.0) (blue), as a function of temperature.

Table S2. Temperature-dependent ^{1}H and ^{7}Li self-diffusion coefficients D (in m^{2}s^{-1}) measured for ChCl:U−LiCl(0), ChCl:U−LiCl(0.8) and ChCl:U−LiCl(1.0). Maximum errors are estimated to be 3% for ^{1}H and 5% for ^{7}Li.

T (K)	ChCl:U−LiCl(0)		ChCl:U−LiCl(0.8)			ChCl:U−LiCl(1.0)		
	U	Ch	U	Ch	Li	U	Ch	Li
298	$3.03{\cdot}10^{-12}$	$2.44{\cdot}10^{-12}$	$1.20{\cdot}10^{-12}$	$1.08{\cdot}10^{-12}$		$1.02{\cdot}10^{-12}$	$8.20{\cdot}10^{-13}$	
303	$5.00{\cdot}10^{-12}$	$3.72{\cdot}10^{-12}$	$1.80{\cdot}10^{-12}$	$1.55{\cdot}10^{-12}$		$1.70{\cdot}10^{-12}$	$1.10{\cdot}10^{-12}$	
308	$7.04{\cdot}10^{-12}$	$4.74{\cdot}10^{-12}$	$2.69{\cdot}10^{-12}$	$2.17{\cdot}10^{-12}$		$2.20{\cdot}10^{-12}$	$1.50{\cdot}10^{-12}$	
313	$1.01{\cdot}10^{-11}$	$6.24{\cdot}10^{-12}$	$3.97{\cdot}10^{-12}$	$2.97{\cdot}10^{-12}$		$2.80{\cdot}10^{-12}$	$2.00{\cdot}10^{-12}$	
318	$1.44{\cdot}10^{-11}$	$8.27{\cdot}10^{-12}$	$5.72{\cdot}10^{-12}$	$3.96{\cdot}10^{-12}$	$2.44{\cdot}10^{-12}$	$4.30{\cdot}10^{-12}$	$2.90{\cdot}10^{-12}$	$1.84{\cdot}10^{-12}$
323	$1.98{\cdot}10^{-11}$	$1.10{\cdot}10^{-11}$	$8.21{\cdot}10^{-12}$	$5.29{\cdot}10^{-12}$	$3.42{\cdot}10^{-12}$	$6.00{\cdot}10^{-12}$	$4.10{\cdot}10^{-12}$	$2.76{\cdot}10^{-12}$
328	$2.66{\cdot}10^{-11}$	$1.45{\cdot}10^{-11}$	$1.13{\cdot}10^{-11}$	$6.85{\cdot}10^{-12}$	$3.93{\cdot}10^{-12}$	$7.37{\cdot}10^{-12}$	$5.10{\cdot}10^{-12}$	$3.27{\cdot}10^{-12}$
333	$3.46{\cdot}10^{-11}$	$1.87{\cdot}10^{-11}$	$1.32{\cdot}10^{-11}$	$8.11{\cdot}10^{-12}$	$5.40{\cdot}10^{-12}$	$1.07{\cdot}10^{-11}$	$6.98{\cdot}10^{-12}$	$4.66{\cdot}10^{-12}$
338	$4.42{\cdot}10^{-11}$	$2.39{\cdot}10^{-11}$	$1.90{\cdot}10^{-11}$	$1.12{\cdot}10^{-11}$	$7.70{\cdot}10^{-12}$	$1.54{\cdot}10^{-11}$	$9.39{\cdot}10^{-12}$	$6.87{\cdot}10^{-12}$
343	$5.47{\cdot}10^{-11}$	$2.98{\cdot}10^{-11}$	$2.51{\cdot}10^{-11}$	$1.44{\cdot}10^{-11}$	$1.01{\cdot}10^{-11}$	$2.01{\cdot}10^{-11}$	$1.20{\cdot}10^{-11}$	$8.82{\cdot}10^{-12}$
348	$7.24{\cdot}10^{-11}$	$3.83{\cdot}10^{-11}$	$3.20{\cdot}10^{-11}$	$1.83{\cdot}10^{-11}$	$1.29{\cdot}10^{-11}$	$2.63{\cdot}10^{-11}$	$1.56{\cdot}10^{-11}$	$1.17{\cdot}10^{-11}$
353	$8.52{\cdot}10^{-11}$	$4.63{\cdot}10^{-11}$	$4.10{\cdot}10^{-11}$	$2.37{\cdot}10^{-11}$	$1.70{\cdot}10^{-11}$	$3.80{\cdot}10^{-11}$	$2.23{\cdot}10^{-11}$	$1.71{\cdot}10^{-11}$
358	$9.87{\cdot}10^{-11}$	$5.45{\cdot}10^{-11}$	$5.04{\cdot}10^{-11}$	$2.91{\cdot}10^{-11}$	$2.12{\cdot}10^{-11}$	$4.79{\cdot}10^{-11}$	$2.76{\cdot}10^{-11}$	$2.04{\cdot}10^{-11}$
363	$1.18{\cdot}10^{-10}$	$6.49{\cdot}10^{-11}$	$6.24{\cdot}10^{-11}$	$3.63{\cdot}10^{-11}$	$2.77{\cdot}10^{-11}$	$5.80{\cdot}10^{-11}$	$3.37{\cdot}10^{-11}$	$2.51{\cdot}10^{-11}$
368	$1.36{\cdot}10^{-10}$	$7.54{\cdot}10^{-11}$	$7.72{\cdot}10^{-11}$	$4.70{\cdot}10^{-11}$	$3.97{\cdot}10^{-11}$	$6.98{\cdot}10^{-11}$	$4.06{\cdot}10^{-11}$	$3.03{\cdot}10^{-11}$
373	$1.58{\cdot}10^{-10}$	$8.92{\cdot}10^{-11}$	$9.26{\cdot}10^{-11}$	$5.60{\cdot}10^{-11}$	$4.43{\cdot}10^{-11}$	$8.37{\cdot}10^{-11}$	$4.83{\cdot}10^{-11}$	$3.65{\cdot}10^{-11}$

Table S3. Parameters obtained from the linear fit of the Arrhenius plot of the diffusion data in the whole temperature range (298-373 K for ^1H, 318-373 K for ^7Li).

Sample	Species	$lnA\ (m^2s^{-1})$	$E_a^{transl}\ (kJmol^{-1})$	R^2
ChCl:U−LiCl(0)	U	-6.8445	48.1	0.990
	Ch	-8.732	44.3	0.998
ChCl:U−LiCl(0.8)	U	-5.7644	53.4	0.996
	Ch	-8.0666	48.1	0.999
	Li	-6.6553	53.2	0.997
ChCl:U−LiCl(1.0)	U	-5.3153	55.1	0.998
	Ch	-6.9686	51.8	0.999
	Li	-6.2722	54.7	0.995

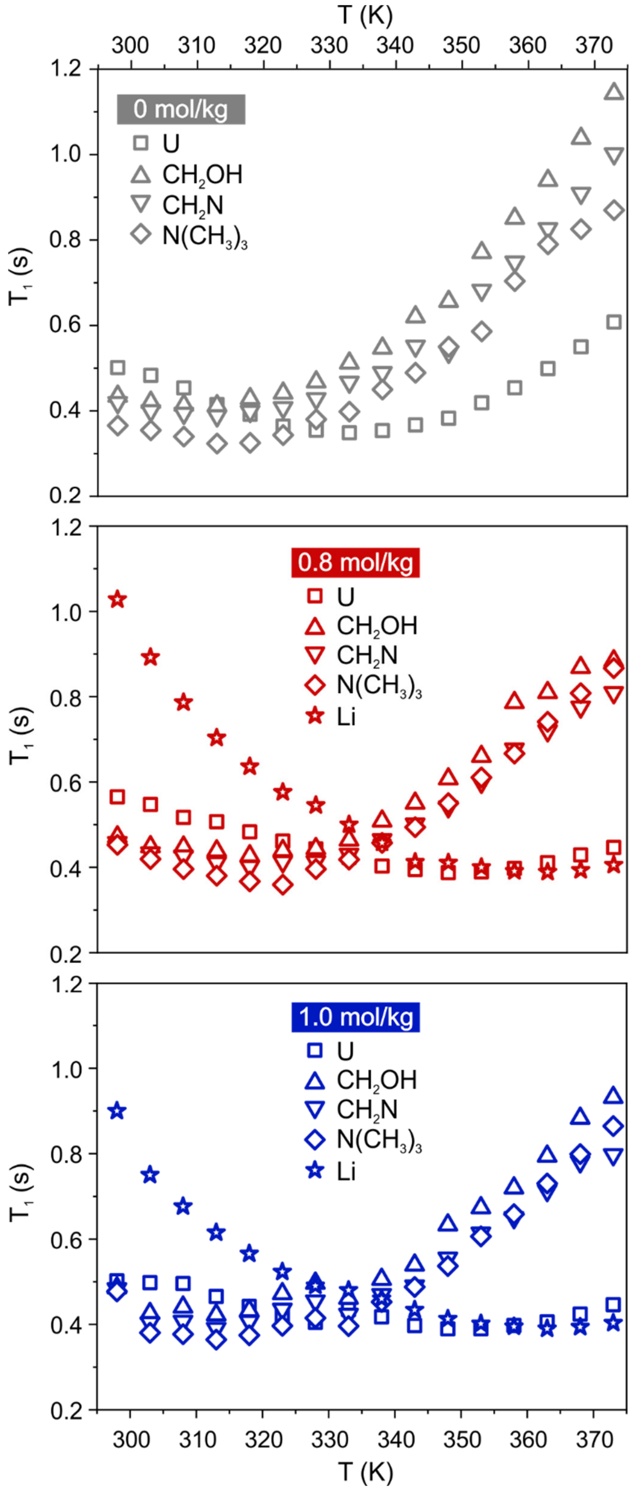

Figure S3. ^{1}H and ^{7}Li T_1 relaxation times of ChCl:U-LiCl(0) (top, grey), ChCl:U-LiCl(0.8) (middle, red), and ChCl:U-LiCl(1.0) (bottom, blue), as a function of temperature. Maximum errors are estimated to be 3%.

Table S4. Best-fit parameters obtained for all proton and lithium nuclei using the BPP model. Maximum errors are estimated to be 15% for τ_0, 5% for E_a^{rot}, 2% for C.

Site	ChCl:U–LiCl(0)			ChCl:U–LiCl(0.8)			ChCl:U–LiCl(1.0)		
	C [10^9] (s^{-2})	E_a^{rot} ($kJmol^{-1}$)	τ_0 (s)	C [10^9] (s^{-2})	E_a^{rot} ($kJmol^{-1}$)	τ_0 (s)	C [10^9] (s^{-2})	E_a^{rot} ($kJmol^{-1}$)	τ_0 (s)
U[a]	6.3	30.6	$3.0 \cdot 10^{-15}$	5.6	28.4	$3.0 \cdot 10^{-14}$	-	-	-
U[b]	6.3	31.1	$2.5 \cdot 10^{-15}$	5.7	28.9	$9.9 \cdot 10^{-15}$	5.7	27.2	$1.7 \cdot 10^{-14}$
CH_2OH	5.4	26.8	$5.9 \cdot 10^{-15}$	5.2	25.6	$1.1 \cdot 10^{-14}$	5.1	24.1	$1.9 \cdot 10^{-14}$
CH_2N	5.7	26.4	$7.5 \cdot 10^{-15}$	5.5	25.5	$1.2 \cdot 10^{-14}$	5.4	24.2	$1.9 \cdot 10^{-14}$
$N(CH_3)_3$	6.7	28.7	$3.1 \cdot 10^{-15}$	5.9	29.0	$3.3 \cdot 10^{-15}$	5.9	27.7	$5.2 \cdot 10^{-15}$
Li	-	-	-	2.2	24.2	$1.7 \cdot 10^{-13}$	2.2	20.0	$6.9 \cdot 10^{-13}$

[a] T_1 curves fitted in the range 308-373 K and 323-373 K for samples ChCl:U-LiCl(0) and ChCl:U-LiCl(0.8), respectively.
[b] T_1 curves fitted in the range 333-373K for all samples.

Table S5. [1]H rotational correlation times τ_C (s) calculated for ChCl:U-LiCl(0) using the BPP model.

T (K)	U*	CH_2OH	CH_2N	$N(CH_3)_3$
298	-	$2.94\cdot10^{-10}$	$3.14\cdot10^{-10}$	$3.33\cdot10^{-10}$
303	-	$2.46\cdot10^{-10}$	$2.64\cdot10^{-10}$	$2.75\cdot10^{-10}$
308	$4.66\cdot10^{-10}$	$2.07\cdot10^{-10}$	$2.23\cdot10^{-10}$	$2.29\cdot10^{-10}$
313	$3.85\cdot10^{-10}$	$1.75\cdot10^{-10}$	$1.89\cdot10^{-10}$	$1.91\cdot10^{-10}$
318	$3.20\cdot10^{-10}$	$1.49\cdot10^{-10}$	$1.61\cdot10^{-10}$	$1.61\cdot10^{-10}$
323	$2.67\cdot10^{-10}$	$1.27\cdot10^{-10}$	$1.38\cdot10^{-10}$	$1.36\cdot10^{-10}$
328	$2.25\cdot10^{-10}$	$1.09\cdot10^{-10}$	$1.19\cdot10^{-10}$	$1.15\cdot10^{-10}$
333	$1.90\cdot10^{-10}$	$9.42\cdot10^{-11}$	$1.03\cdot10^{-10}$	$9.84\cdot10^{-11}$
338	$1.61\cdot10^{-10}$	$8.17\cdot10^{-11}$	$8.92\cdot10^{-11}$	$8.44\cdot10^{-11}$
343	$1.38\cdot10^{-10}$	$7.11\cdot10^{-11}$	$7.78\cdot10^{-11}$	$7.27\cdot10^{-11}$
348	$1.18\cdot10^{-10}$	$6.21\cdot10^{-11}$	$6.81\cdot10^{-11}$	$6.29\cdot10^{-11}$
353	$1.02\cdot10^{-10}$	$5.45\cdot10^{-11}$	$5.99\cdot10^{-11}$	$5.46\cdot10^{-11}$
358	$8.78\cdot10^{-11}$	$4.79\cdot10^{-11}$	$5.28\cdot10^{-11}$	$4.77\cdot10^{-11}$
363	$7.62\cdot10^{-11}$	$4.23\cdot10^{-11}$	$4.68\cdot10^{-11}$	$4.17\cdot10^{-11}$
368	$6.64\cdot10^{-11}$	$3.75\cdot10^{-11}$	$4.15\cdot10^{-11}$	$3.67\cdot10^{-11}$
373	$5.81\cdot10^{-11}$	$3.34\cdot10^{-11}$	$3.70\cdot10^{-11}$	$3.23\cdot10^{-11}$

* T_1 curve fitted in the range 308-373 K.

Table S6. ^1H and ^7Li rotational correlation times τ_C (s) calculated for ChCl:U-LiCl(0.8) using the BPP model.

T (K)	U*	CH_2OH	CH_2N	$N(CH_3)_3$	Li
298		$3.41 \cdot 10^{-10}$	$3.63 \cdot 10^{-10}$	$4.01 \cdot 10^{-10}$	$2.97 \cdot 10^{-9}$
303		$2.88 \cdot 10^{-10}$	$3.06 \cdot 10^{-10}$	$3.30 \cdot 10^{-10}$	$2.53 \cdot 10^{-9}$
308		$2.44 \cdot 10^{-10}$	$2.60 \cdot 10^{-10}$	$2.74 \cdot 10^{-10}$	$2.16 \cdot 10^{-9}$
313		$2.08 \cdot 10^{-10}$	$2.21 \cdot 10^{-10}$	$2.29 \cdot 10^{-10}$	$1.86 \cdot 10^{-9}$
318		$1.78 \cdot 10^{-10}$	$1.90 \cdot 10^{-10}$	$1.92 \cdot 10^{-10}$	$1.61 \cdot 10^{-9}$
323	$3.93 \cdot 10^{-10}$	$1.53 \cdot 10^{-10}$	$1.63 \cdot 10^{-10}$	$1.62 \cdot 10^{-10}$	$1.40 \cdot 10^{-9}$
328	$3.40 \cdot 10^{-10}$	$1.32 \cdot 10^{-10}$	$1.41 \cdot 10^{-10}$	$1.37 \cdot 10^{-10}$	$1.22 \cdot 10^{-9}$
333	$2.96 \cdot 10^{-10}$	$1.15 \cdot 10^{-10}$	$1.23 \cdot 10^{-10}$	$1.17 \cdot 10^{-10}$	$1.06 \cdot 10^{-9}$
338	$2.58 \cdot 10^{-10}$	$1.00 \cdot 10^{-10}$	$1.07 \cdot 10^{-10}$	$1.00 \cdot 10^{-10}$	$9.35 \cdot 10^{-10}$
343	$2.26 \cdot 10^{-10}$	$8.79 \cdot 10^{-11}$	$9.39 \cdot 10^{-11}$	$8.63 \cdot 10^{-11}$	$8.25 \cdot 10^{-10}$
348	$1.99 \cdot 10^{-10}$	$7.72 \cdot 10^{-11}$	$8.26 \cdot 10^{-11}$	$7.46 \cdot 10^{-11}$	$7.30 \cdot 10^{-10}$
353	$1.76 \cdot 10^{-10}$	$6.81 \cdot 10^{-11}$	$7.29 \cdot 10^{-11}$	$6.47 \cdot 10^{-11}$	$6.49 \cdot 10^{-10}$
358	$1.56 \cdot 10^{-10}$	$6.03 \cdot 10^{-11}$	$6.45 \cdot 10^{-11}$	$5.64 \cdot 10^{-11}$	$5.78 \cdot 10^{-10}$
363	$1.38 \cdot 10^{-10}$	$5.36 \cdot 10^{-11}$	$5.74 \cdot 10^{-11}$	$4.93 \cdot 10^{-11}$	$5.17 \cdot 10^{-10}$
368	$1.23 \cdot 10^{-10}$	$4.77 \cdot 10^{-11}$	$5.11 \cdot 10^{-11}$	$4.33 \cdot 10^{-11}$	$4.63 \cdot 10^{-10}$
373	$1.10 \cdot 10^{-10}$	$4.27 \cdot 10^{-11}$	$4.57 \cdot 10^{-11}$	$3.81 \cdot 10^{-11}$	$4.17 \cdot 10^{-10}$

* T_1 curve fitted in the range 323-373 K.

Table S7. ^1H and ^7Li rotational correlation times τ_C (s) calculated for ChCl:U-LiCl(1.0) using the BPP model.

T (K)	U*	CH_2OH	CH_2N	$N(CH_3)_3$	Li
298		$3.11 \cdot 10^{-10}$	$3.33 \cdot 10^{-10}$	$3.73 \cdot 10^{-10}$	$2.31 \cdot 10^{-9}$
303		$2.65 \cdot 10^{-10}$	$2.84 \cdot 10^{-10}$	$3.10 \cdot 10^{-10}$	$2.02 \cdot 10^{-9}$
308		$2.27 \cdot 10^{-10}$	$2.43 \cdot 10^{-10}$	$2.60 \cdot 10^{-10}$	$1.77 \cdot 10^{-9}$
313		$1.95 \cdot 10^{-10}$	$2.09 \cdot 10^{-10}$	$2.18 \cdot 10^{-10}$	$1.56 \cdot 10^{-9}$
318		$1.69 \cdot 10^{-10}$	$1.80 \cdot 10^{-10}$	$1.85 \cdot 10^{-10}$	$1.39 \cdot 10^{-9}$
323		$1.47 \cdot 10^{-10}$	$1.56 \cdot 10^{-10}$	$1.57 \cdot 10^{-10}$	$1.23 \cdot 10^{-9}$
328		$1.28 \cdot 10^{-10}$	$1.36 \cdot 10^{-10}$	$1.34 \cdot 10^{-10}$	$1.10 \cdot 10^{-9}$
333	$3.37 \cdot 10^{-10}$	$1.12 \cdot 10^{-10}$	$1.19 \cdot 10^{-10}$	$1.15 \cdot 10^{-10}$	$9.84 \cdot 10^{-10}$
338	$2.89 \cdot 10^{-10}$	$9.84 \cdot 10^{-11}$	$1.05 \cdot 10^{-10}$	$9.94 \cdot 10^{-11}$	$8.84 \cdot 10^{-10}$
343	$2.49 \cdot 10^{-10}$	$8.68 \cdot 10^{-11}$	$9.25 \cdot 10^{-11}$	$8.61 \cdot 10^{-11}$	$7.96 \cdot 10^{-10}$
348	$2.15 \cdot 10^{-10}$	$7.69 \cdot 10^{-11}$	$8.19 \cdot 10^{-11}$	$7.49 \cdot 10^{-11}$	$7.20 \cdot 10^{-10}$
353	$1.87 \cdot 10^{-10}$	$6.83 \cdot 10^{-11}$	$7.27 \cdot 10^{-11}$	$6.54 \cdot 10^{-11}$	$6.52 \cdot 10^{-10}$
358	$1.63 \cdot 10^{-10}$	$6.09 \cdot 10^{-11}$	$6.48 \cdot 10^{-11}$	$5.73 \cdot 10^{-11}$	$5.93 \cdot 10^{-10}$
363	$1.42 \cdot 10^{-10}$	$5.45 \cdot 10^{-11}$	$5.79 \cdot 10^{-11}$	$5.04 \cdot 10^{-11}$	$5.40 \cdot 10^{-10}$
368	$1.25 \cdot 10^{-10}$	$4.89 \cdot 10^{-11}$	$5.19 \cdot 10^{-11}$	$4.45 \cdot 10^{-11}$	$4.94 \cdot 10^{-10}$
373	$1.10 \cdot 10^{-10}$	$4.40 \cdot 10^{-11}$	$4.67 \cdot 10^{-11}$	$3.94 \cdot 10^{-11}$	$4.52 \cdot 10^{-10}$

* T_1 curve fitted in the range 333-373 K.

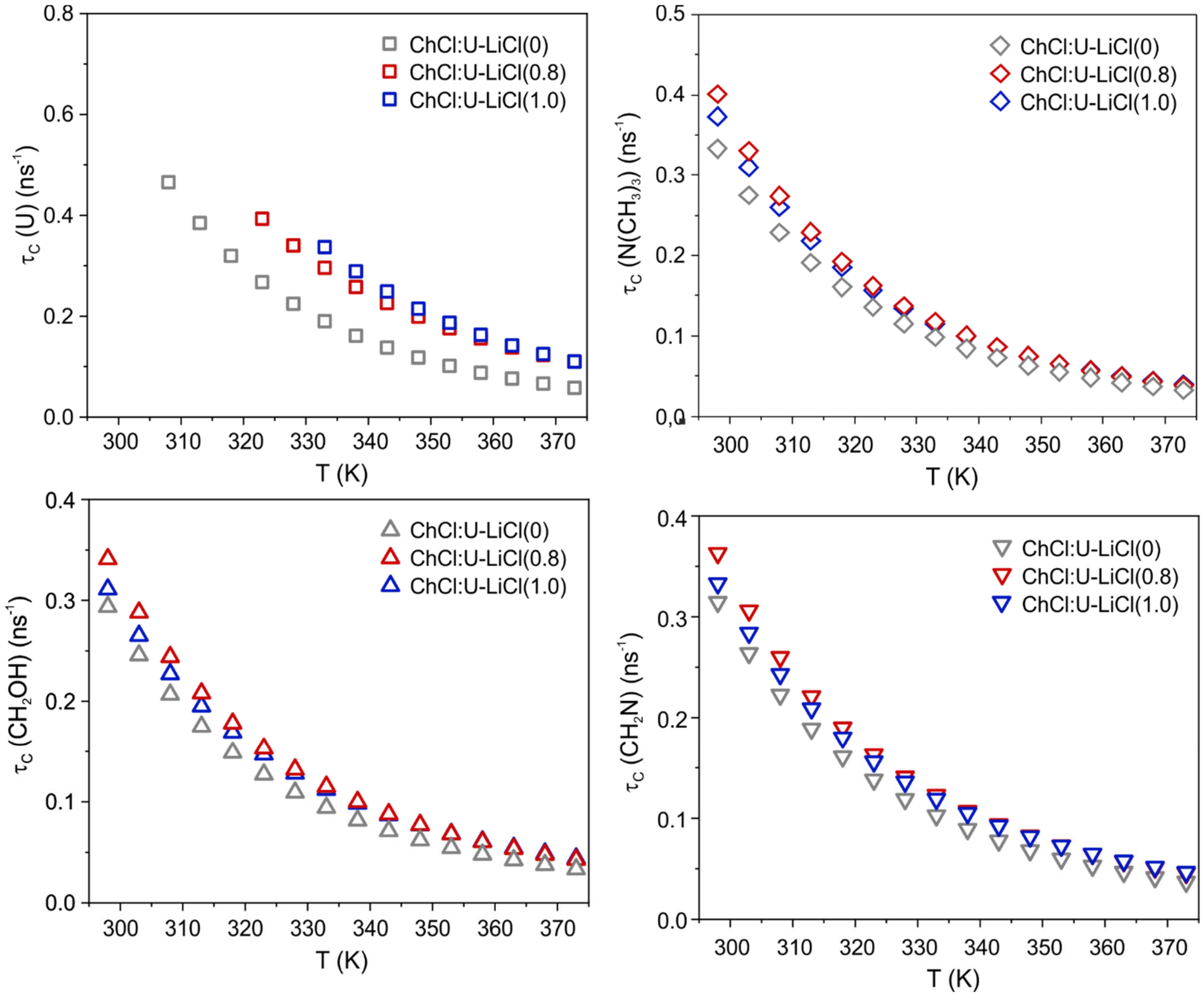

Figure S4. ^{1}H correlation time τ_C calculated as a function of temperature in the samples ChCl:U-LiCl(0) (grey), ChCl:U-LiCl(0.8) (red), and ChCl:U-LiCl(1.0) (blue) for U protons (squares, top, left), $N(CH_3)_3$ protons of Ch (diamonds, top, right), CH_2OH protons of Ch (triangles, bottom, left) and CH_2N protons of Ch (inverted triangles, bottom, right).

References

1. Smith, E.L.; Abbott, A.P.; Ryder, K.S. Deep Eutectic Solvents (DESs) and Their Applications. *Chem. Rev.* **2014**, *114*, 11060–11082.
2. Abranches, D.O.; Martins, M.A.R.; Silva, L.P.; Schaeffer, N.; Pinho, S.P.; Coutinho, J.A.P. Phenolic hydrogen bond donors in the formation of non-ionic deep eutectic solvents: The quest for type v des. *Chem. Commun.* **2019**, *55*, 10253–10256.
